# A Pilot Study on Developed Shoes That Enhance Gait Parameters Without Increasing Muscle Activity

**DOI:** 10.1155/abb/5587738

**Published:** 2024-12-14

**Authors:** Teppei Abiko, Shin Murata, Yoshihiro Kai, Hideki Nakano, Masashi Sakamoto, Keita Suzuki, Dai Matsuo, Michio Kawaguchi

**Affiliations:** ^1^Department of Physical Therapy, Faculty of Health Sciences, Kyoto Tachibana University, Kyoto, Japan; ^2^ASICS Trading Company Limited, Kobe, Hyogo, Japan

## Abstract

This pilot study investigated the potential of a newly developed shoe design to improve gait parameters without altering muscle activity in healthy women. The shoe design features a V-shaped heel and a high-elasticity midsole, which are intended to enhance stability during heel contact and promote efficient load transfer throughout the gait cycle. Ten study participants underwent a randomized crossover design, wearing developed and general shoes during the trials. Spatiotemporal gait data and muscle activity were measured to assess the impact of the shoe design developed on gait efficiency. Significant improvements in gait speed, step and stride length, and swing time were observed with the developed shoes, indicating improved gait efficiency. Importantly, these improvements were achieved without significant changes in muscle activity, suggesting that the developed shoe design improves gait efficiency without increasing muscle workload. Considering the limitations of the small sample size and the exploratory nature of this pilot study, further research with a larger cohort is necessary to validate these preliminary findings.

**Trial Registration:** Clinical Trial Registry identifier: UMIN000054260

## 1. Introduction

Maintaining and improving walking ability is crucial for promoting independent living and enhancing the quality of life, especially in older adults [1]. Gait speed is a key indicator of motor function [2], and a decline in walking speed is associated with aging and an increased risk of mortality [3]. For example, a study by Studenski et al. [2] reported a 12% reduction in mortality risk for every 0.1 m/s increase in walking speed among elderly individuals. Additionally, slower gait speed is linked to cognitive decline in patients with coronary artery disease (CAD) [4], highlighting its broader importance for health outcomes.

Several biomechanical factors, such as stride length, cadence, joint kinematics, and muscle activation, influence walking speed and efficiency [5–13]. Studies show that longer strides are correlated with faster walking speeds, while shorter strides can reduce walking efficiency [7, 8]. In response to these challenges, shoe designs that enhance gait efficiency by improving these parameters have gained attention.

Energy-returning shoes, for instance, use advanced materials to store and release energy during walking, potentially reducing muscle activity, and improving overall performance [14–16]. Conversely, rocker shoes, like the Masai Barefoot Technology (MBT), have been shown to improve posture and reduce joint motion, although they can lead to shorter stride lengths due to instability [17–22]. Regarding the cause of the shorter stride length, it was mentioned that the instability caused by the round soles may lead to a more cautious gait pattern.

In this context, the development of shoes that can enhance gait performance while maintaining stability is crucial. This pilot study aimed to test a new shoe design that combines a rocker function with a stable support base. The study focused on comparing the spatiotemporal gait parameters and muscle activities of healthy participants walking in the developed shoes versus general shoes. Although this study was limited by the small sample size and testing on young, healthy women, it provides preliminary insights that can guide future studies on more diverse populations, including older adults with gait impairments.

## 2. Materials and Methods

### 2.1. Subjects

Ten healthy women with a mean age of 20.7 ± 0.7 years, a mean height of 157.5 ± 4.7 cm, a mean body weight of 51.4 ± 6.2 kg, and a mean body mass index of 23.0 ± 2.5 kg/m^2^ whose shoe size was 24.0 cm participated in this study. All participants were recruited from Kyoto Tachibana University. Only female participants were recruited due to differences in sex in muscle strength and electromyographic (EMG) activity [23–26]. The exclusion criteria were as follows: individuals with foot pain within the previous 6 months, those with previous foot surgery, those with congenital or acquired foot deformities, and those with any other disabilities that affect gait. None of the participants had cardiovascular, neurological, or musculoskeletal diseases. The study was conducted in accordance with the ethical standards outlined in the Declaration of Helsinki and was approved by the local Institutional Ethics Committee (Approval Number: 21-26, Date of approval: August 21, 2021). Prior to participation, all subjects were provided with a detailed explanation of the study's purpose, procedures, potential risks, and benefits. Written informed consent was obtained from all participants, who were informed of their right to withdraw from the study at any time without penalty or need to provide a reason. Confidentiality and anonymity of all personal data were strictly maintained throughout the study. Additionally, the study followed the ethical principles established by the STROBE guidelines for observational studies. No external funding or conflicts of interest that could have influenced the conduct of the study were present.

### 2.2. Procedure

A randomized crossover design was used, and participants were randomly assigned to two sequences of different shoes using random numbers generated using Microsoft Excel 2010. In Sequence 1, the participants wore the developed shoes first, whereas, in Sequence 2, the participants wore general shoes first. After 1 week, both groups changed shoes; this switch was called the washout period. This design was chosen to minimize potential biases, such as fatigue, order effects, and learning effects, by allowing each participant to serve as their own control. The 1-week washout period was implemented to reduce carryover effects from the previous condition.

**Figure 1:**
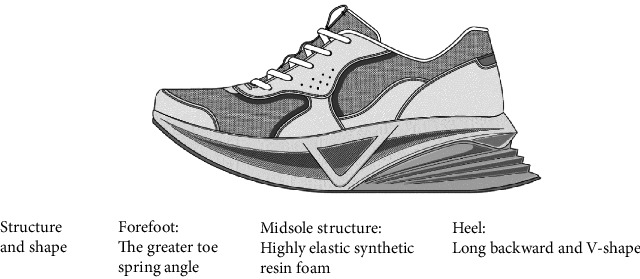
Schematic of the developed shoes.

**Figure 2:**
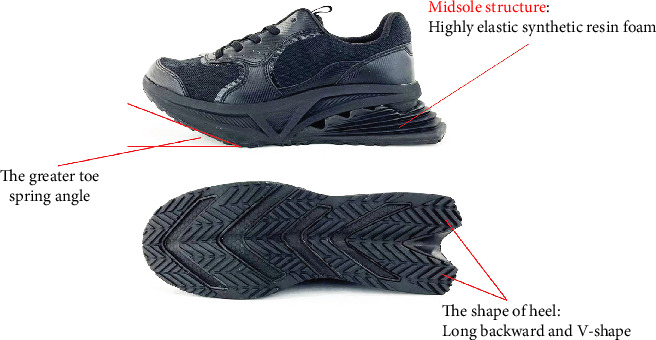
The developed shoes.

**Figure 3:**
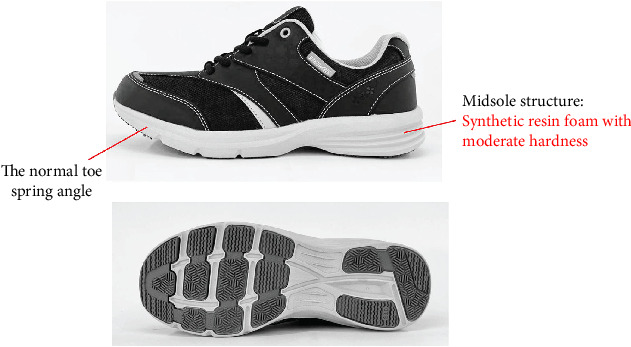
The general shoes; RAKUWALK BasicAid (Asics Corp.).

The shoes developed have several unique structural and shape features. The main structural characteristic of the developed shoes was that the midsole is made of high-elasticity synthetic resin foam (Figures 1 and 2). The midsole consists of multiple layers, including a high-elasticity section that uses materials such as EVA, TPU, or foam rubber. It helps absorb the impact of heel contact and generate repulsive force, thus promoting load transfer to the forefoot. With respect to shape, the developed shoes have two distinctive characteristics. First, the heel is long toward the back and has a V shape. The purpose of this shape is to increase the stability from heel contact to midstance and to promote heel contact and smooth forward movement of the center of pressure with a wide base of support. The heel's V-shape, the rearward extension, and the wide base of support help prevent lateral instability and ensure that the load is distributed centrally, thereby reducing unnecessary side-to-side movement. Second, the toe spring angle is about 15° and greater to enhance the propulsive force of the forward movement of the center of gravity from midstance to heel off. RAKUWALK (Asics Corp), a marketable shoe brand, was used as general shoes (Figure 3). The midsole of general shoes is made of moderate hard synthetic resin foam (Figure 2). Regarding the shape of the forefoot, general shoes have a smaller toe spring angle than developed shoes.

The subjects walked on an 8-m walkway using the developed and general shoes. At each walking session, they performed one familiarization trial and two experimental trials, during which the temporal and spatial gait parameters and the EMG results were collected concurrently. The walking trials were conducted in a university corridor with a concrete floor, which is commonly used in educational facilities for its durability and stability. The area was illuminated by standard fluorescent lighting, providing an average illuminance of ~200 lux.

### 2.3. Measurements and Data Analysis

The spatiotemporal gait data were measured using the OptoGait photoelectric cell system (OptoGait, Microgate, Bolzano, Italy), which consists of light-transmitting and light-receiving bars [27]. Each bar is 1 m long and consists of 100 light-emitting diodes that continuously emit light to an opposing bar. As subjects walk between two parallel bars positioned on the ground, their feet block the transmission and reception of light, allowing the system to determine the distance and duration and automatically calculate spatiotemporal parameters. The OptoGait photoelectric cell system was connected to a personal computer through an interface unit and OptoGait software was used to extract data at a sampling frequency of 1000 Hz. The mean walking speed (m/s), cadence (steps/min), step length (cm), stride length (cm), stance time (s), swing time (s), single-stance time (s), and double-stance time (s) were measured during the two experimental sessions.

Muscle activities of the bellies of the right tibialis anterior (TA), the right gastrocnemius medialis (GM), and the right gastrocnemius lateralis (GL) were measured using a surface electromyogram (TeleMyoG2; Noraxon Inc., USA). Electromyogram surface electrodes (Blue sensor; Ambu Inc., Denmark) were attached according to SENIAM after hair removal and skin abrasion [28]. The electrodes were placed at specific locations, such as the TA at one-third of the line between the tip of the fibula and the tip of the medial malleolus, the GM at the most prominent bulge of the muscle, and the GL at one-third of the line between the head of the fibula and the heel. An interelectrode distance of 2 cm was maintained from center to center, and the reference electrode was fixed to the head of the right fibula. The signals were sampled at 1000 Hz and bandpass filtered (20–500 Hz) using analysis software (MyoResearch XP; Noraxon Inc., USA) and transferred to a personal computer. Isometric maximum voluntary contractions (MVCs) were performed twice for all muscles recorded against manual resistance for 3 s for normalization. TA was measured while generating maximal isometric dorsiflexion in the sitting position against plantar flexion resistance, and GM and GL were tested while maintaining a raised right heel standing in the extended knee position against shoulder pressure. The middle 1 s of the entire 3 s measurement of the MVC data was selected, after which the mean values of each muscle were calculated [15].

To obtain integrated EMG (IEMG) data in the stance and swing phases, the EMG signals were subjected to full-wave rectification and normalized to 100% of the gait cycle time using OptoGait data synchronized with the EMG system. By synchronizing the EMG system with OptoGait data, the stance and swing phases were clearly distinguished. Then, the % IEMG data of the stance and swing phases were normalized to the MVC values of each muscle. The mean values of four steps for the two experimental sessions were calculated for representative data [15]. To minimize potential biases, the analysis of spatiotemporal gait data and the electromyography data was conducted with masking in place for the settings of both the developed shoes and the general shoes.

For statistical analysis, means and standard deviations are used to present the results. The Mann–Whitney *U* test was used to compare characteristics between groups. A general linear mixed model was developed to estimate the effects of the shoes developed (treatment effect), which included fixed effects of the shoes developed, sequence (order of shoes), and period (order of time). The inclusion of the sequence and period effects in the model was to account for possible carryover and familiarization effects, respectively. *p*-Values < 0.05 were used to denote statistical significance and all statistical analyzes were performed using Statistical Package for the Social Sciences, version 26.0 (IBM Corp., Armonk, NY, USA).

## 3. Results and Discussion

No significant differences were observed in any of the descriptive variables between the groups (Table 1). The analysis showed no sequence or period effects for any of the parameters examined. The mean values of the gait parameters for the developed and general shoes are outlined in Table 2. Accordingly, those wearing the developed shoes had a faster walking speed (*p*=0.047), a longer step (*p*=0.023), and a longer stride (*p*=0.018) than those wearing general shoes (Table 2). Additionally, those wearing the developed shoes had significantly shorter swing time (*p* < 0.001), whereas no differences in cadence and stance time were observed between the two groups. The mean % IEMG values for general and developed shoes are summarized in Table 3. During the stance phase, those wearing the developed shoes had significantly higher % IEMG for the TA than those wearing the general insoles (TA: *p*=0.01), while no significant differences were observed in the mean % IEMG during the swing phase.

We investigated the immediate effects of the developed and general shoes on gait parameters and muscle activities while healthy women walked. Gait speed, step length, stride length, and swing time among the gait parameters under study were significantly improved in those who wore developed shoes compared to those who wore general shoes. Muscle activity was similar between the two groups of shoes, except for TA during the stance phase. These results suggest that the developed shoe design can improve gait efficiency, allowing people to walk faster without increasing their muscle activity.

Rocker shoes, such as the MBT, are assumed to provide a smooth shift of the sagittal center of gravity, an increase in walking speed, and an increase in stride length due to their shape. However, as the result of MBT study found that stride length decreased, while other spatial-temporal parameters, such as gait velocity, remained unchanged in the general adult population [29]. Thus, we developed shoes with a rocker function but with a stable and wide support base, which introduces a novel and innovative method to improve gait efficiency. In the developed shoe, the rear foot was made in a V-shaped form and was significantly extended backward to increase the supportive base surface of the rear foot to improve stability during walking. Therefore, the single-stance time was shortened and the dual-stance time was extended in the developed shoe group compared to those in the ordinary shoe group, and it can be judged that walking became more stable. This stability is thought to allow the walking pattern to change to one in which the rocker function of the shoes can be used.

To obtain the rocker function of the developed shoes, a high-elasticity synthetic resin foam was used for the midsole. The use of a cushioned material is thought to encourage a forward shift of the center of gravity by sinking and transforming it into a rounded form. Furthermore, this structure allows the heel to absorb the impact [30] and generate a repulsive force during landing and push-off, respectively. This feature may facilitate a more efficient energy transfer and a smoother gait cycle. Regarding the forefoot, the shape of the sole enhances the function of the forefoot locker, which can increase walking speed and stride length [31]. On the contrary, general shoes have a midsole made of moderate hard synthetic resin foam without the highly elastic material in the heel. The shape of the forefoot also has a lower spring angle of the toes than those of developed shoes. These characteristics may result in a less efficient gait cycle, and improvements in walking speed and stride length may be minimal [32]. Therefore, the distinctive structures and features of the shoes developed may improve gait efficiency and walking performance.

The results of the EMG data revealed that participants wearing newly developed shoes exhibited a significantly higher % IEMG for the TA muscle during the stance phase than those wearing general shoes. These findings are consistent with those of the study by Hof et al. [33]., which demonstrated that TA muscle activity tended to increase proportionally with gait speed. It is inferred in this study that the observed increase in TA muscle activity could be attributed to the increased walking speed facilitated by the developed shoes. Moreover, the observed increase was exclusive to the stance phase. This could be attributed to the soft and unstable material of the shoe soles in the developed shoes. It was plausible that these characteristics led to an adaptive response in which balance during walking was maintained through upregulation of TA muscle activity [34]. Romkes, Rudmann, and Brunner [19] reported that the muscle activity in the lower limbs increased when using MBT rocker shoes, which they attributed to the instability of the rocker sole causing co-contraction of the ankle muscles. In contrast, our developed shoes did not elicit the same effect as MBT shoes, as GM and GL muscle activity remained comparable to that observed with general shoes. This can likely be explained by the wider support base of the developed shoes, which may have provided greater stability. Consequently, it can be inferred that the developed shoes did not introduce significant instability, thereby allowing participants to walk without an increase in co-contraction or muscle activity.

Participants wearing the developed shoes showed significant improvements in gait speed, step length, stride length, and swing time compared to those wearing general shoes. However, the mean % IEMG was similar between the two shoes, except for the TA during the stance phase. This result is notably different from previous research on rocker shoes, which have generally demonstrated no significant change in gait speed and have even reported increased muscle activity in the lower extremities [19, 29]. Our results suggest that the shoe design developed improves gait efficiency by allowing participants to walk faster without significantly increasing muscle activity of GM and GL. The highly elastic material in the heel of the midsole may have contributed to this effect by absorbing the impact and generating a repulsive force during landing and push-off, respectively. This feature may have facilitated more efficient energy transfer and muscle activation during gait. Furthermore, the fact that the IEMGs of both groups were almost the same suggests that the developed shoes have inhibited unwanted muscle activity that could have caused fatigue and reduced walking efficiency. Therefore, the shoe design developed seemed to have improved gait efficiency by allowing participants to walk faster without increasing their muscle workload.

Several limitations of this study should be acknowledged. First, the sample size was limited due to the inability to produce multiple shoe sizes, constrained by both cost and time considerations in assessing the effectiveness of the developed shoes. Furthermore, the study used only a single shoe size and involved exclusively female participants, restricting the generalizability of the results. Consequently, the findings may not extend to other populations, such as males, individuals with different foot sizes, or those with gait impairments. Second, the study lacked long-term follow-up to determine whether the observed improvements in gait parameters are sustainable. Short-term changes in gait speed and stride length may diminish without continued use of the developed shoes, emphasizing the need for long-term studies to assess both durability and potential adverse effects. In particular, there is a potential risk associated with the V-shaped heel design, which may cause heel collisions with stair nosing or risers when descending stairs, increasing the risk of falls. It is therefore essential to strike a balance, ensuring the heel is not excessively long while still facilitating smooth weight transfer from heel strike to forward movement. Future research should involve a larger and more diverse sample, including older adults and individuals with mobility impairments, to assess the broader applicability of these findings.

## 4. Conclusions

The findings of this pilot study showed the potential of footwear designs as an intervention to improve gait parameters without increasing muscle activity in a younger population. These findings suggest that the developed shoe design may enhance gait efficiency. However, further research is required to confirm these findings and to determine the short- and long-term effects of the developed shoes on gait parameters in diverse populations.

## Figures and Tables

**Table 1:** Participant characteristics based on the order of intervention.

Characteristics	Sequence 1	Sequence 2	All participants
	(*n* = 5)	(*n* = 5)	(*n* = 10)
Age (years)	20.4 ± 0.5	21.0 ± 0.7	20.7 ± 0.7
Height (cm)	160.0 ± 4.9	155.0 ± 3.2	157.5 ± 4.7
Weight (kg)	54.2 ± 5.4	48.6 ± 6.2	51.4 ± 6.2
BMI	18.9 ± 6.3	20.3 ± 3.0	23.0 ± 2.5

**Table 2:** Treatment effect of gait parameters between developed and general shoes.

Gait parameters	Developed shoes	General shoes	Difference	*p*-Value
Walking speed (m/s)	1.46 ± 0.14	1.39 ± 0.16	0.06 ± 0.08	0.047
Cadence (steps/min)	119.75 ± 3.15	118.26 ± 3.97	1.49 ± 3.06	0.160
Step length (cm)	73.02 ± 6.01	70.45 ± 5.98	2.57 ± 2.78	0.023
Stride length (cm)	143.23 ± 12.99	135.36 ± 8.68	7.87 ± 8.17	0.018
Stance time (s)	0.62 ± 0.02	0.60 ± 0.03	0.02 ± 0.02	0.152
Swing time (s)	0.39 ± 0.01	0.42 ± 0.01	−0.03 ± 0.01	*p* < 0.001
Single-stance time (s)	0.39 ± 0.01	0.42 ± 0.01	−0.03 ± 0.01	*p* < 0.001
Double-stance time (s)	0.23 ± 0.02	0.19 ± 0.03	0.04 ± 0.02	*p* < 0.001

**Table 3:** Treatment effect of % integrated electromyography values for each muscle during the stance and swing phases between the developed and general shoes.

Gait phase	Muscle	Developed shoes	General shoes	Difference	*p*-Value
Stance phase	TA	15.91 ± 3.95	12.34 ± 2.81	3.57 ± 3.20	0.010
	GM	40.96 ± 11.46	35.92 ± 10.78	5.04 ± 8.77	0.125
	GL	36.54 ± 20.65	34.60 ± 20.70	1.95 ± 4.50	0.215

Swing phase	TA	20.57 ± 8.42	21.16 ± 6.97	−0.59 ± 3.17	0.521
	GM	17.11 ± 13.91	14.63 ± 14.13	2.48 ± 10.20	0.487
	GL	12.44 ± 10.24	9.65 ± 8.18	2.78 ± 5.03	0.130

Abbreviations: GL, gastrocnemius lateralis; GM, gastrocnemius medialis; TA, tibialis anterior.

## Data Availability

The data that support the findings of this study are available from the corresponding author upon reasonable request.
